# HutZ is required for efficient heme utilization and contributes to the pathogenicity of *Avibacterium paragallinarum*

**DOI:** 10.1128/spectrum.03979-22

**Published:** 2023-09-28

**Authors:** Caiyun Huo, Lijiao Jiao, Guiping Li, Donghai Li, Wutong Lin, Yingjian Sun, Huiling Sun

**Affiliations:** 1 Beijing Key Laboratory for Prevention and Control of Infectious Diseases in Livestock and Poultry, Institute of Animal Husbandry and Veterinary Medicine, Beijing Academy of Agriculture and Forestry Sciences, Beijing, China; 2 Animal Science and Technology College, Beijing University of Agriculture, Beijing, China; University of Adelaide, Roseworthy, South Australia, Australia

**Keywords:** *Avibacterium paragallinarum*, HutZ, iron homeostasis, heme utilization, pathogenicity

## Abstract

*Avibacterium paragallinarum* is the pathogen that causes infectious coryza, a highly contagious respiratory disease that brings a serious threat to chickens. Heme utilization systems play an important role in bacterial adversity adaptation and pathogenicity, and our previous report found the presence of heme utilization (HutZ) in *Av. paragallinarum*. However, little is known about the function of HutZ in *Av. paragallinarum*. In this study, the HutZ mutant strain of *Av. paragallinarum* was successfully developed and identified by PCR and western blot analysis. Mutation of HutZ significantly retards bacterial growth under reduced iron conditions, indicating the regulatory role of HutZ on growth and iron acquisition. Notably, the HutZ mutant strain had slower growth than the wild-type strain when heme was provided as the sole source of iron; thus, HutZ is crucial for heme utilization in *Av. paragallinarum*. Moreover, the HutZ mutant strain exhibited a markedly compromised tolerance to acid stress compared to the wild-type strain. Pathogenicity analysis showed that mutation of HutZ significantly weakened the ability of bacteria to invade and reproduce in host macrophage cells *in vitro*. Furthermore, the HutZ mutation could significantly decrease the bacterial virulence in chickens, which displayed lower morbidity and milder clinical symptoms. Hence, this is the first study to demonstrate in-depth the essential roles of HutZ on iron homeostasis and pathogenesis of *Av. paragallinarum*, which provides novel insight into advances of new prophylactic vaccines against this kind of bacteria.

**Importance:**

Heme utilization (HutZ) protein has been characterized as an important heme-degrading enzyme that is critical for the cleavage of heme to biliverdin *via* verdoheme and can release iron to be used by bacteria. The interaction between HutZ and *Av. paragallinarum* is still unknown. Here, we unraveled the role of HutZ on the growth, iron acquisition, heme utilization, and resistance to acidic stress in *Av. paragallinarum*. We also uncovered the importance of HutZ for the success of *Av. paragallinarum* infection and provided new clues to the pathogenesis strategies of this organism. This work constitutes a relevant step toward an understanding of the role of HutZ protein as a master virulence factor. Therefore, this study is of great importance for understanding the mechanisms underlying *Av. paragallinarum* virulence and may contribute to therapeutic applications.

## INTRODUCTION

Infectious coryza (IC), caused by Gram-negative bacterium *Avibacterium paragallinarum*, is an acute respiratory disease of poultry that causes enormous economic losses by inducing growth retardation in broilers and impairing egg production (1). The most common clinical signs in chickens infected with *Av. paragallinarum* include facial edema, nasal exudates, sneezing, blindness, and conjunctivitis. The organism can be serotyped by two schemes, namely, the Page and Kume schemes, modified by Blackall et al. (2), which recognizes three serogroups (A, B, and C) and nine serovars (A1-A4, B1, and C1-4) (2). The presence of *Av. paragallinarum* has been reported in many countries, such as South Africa, Mexico, Peru, Indonesia, India, the UK, Japan, Korea, China, and the USA, in recent years (3 – 12). In South Africa, it is considered one of the most serious diseases, with C3 being the predominant serovar (13). In China, serovars A, C, and B were identified for the first time in 1987, 1995, and 2003, respectively (9, 14, 15). Recently, IC outbreaks have been reported in multiple states in the USA, and IC is now considered an emerging respiratory disease of chickens in the northeastern USA, such as Pennsylvania, Delaware, and Maryland (16, 17). Due to the worldwide distribution of *Av. paragallinarum* and a major cause of significant economic losses to the poultry industry, it is important for the development of novel broad-spectrum prophylactic tools against *Av. paragallinarum*. To date, the molecular mechanisms of colonization, growth, and virulence of *Av. paragallinarum* in the chicken upper respiratory tract are still poorly understood.

Iron is an essential micronutrient for most bacteria, which plays essential roles in bacterial colonization, growth, and virulence. Unfortunately, bacteria encounter an extremely low iron milieu, because the majority of iron is sequestered in iron- and heme-containing proteins (18). Therefore, bacteria have evolved sophisticated iron-acquisition systems to scavenge iron from their surroundings (siderophores, hemophores, or host-molecule-binding proteins) to ensure adequate supplies (19). Remarkably, the availability of free iron is strongly limited in vertebrate hosts, causing insufficient iron acquisition by siderophores, especially for pathogenic bacteria (20). Thus, the heme utilization system has been well developed in bacteria to better obtain iron, which is considered a common mechanism employed by pathogens. For example, heme is preferred as an iron source over ferrous iron for some Gram-positive bacteria such as *Staphylococcus aureus* and *Streptococcus pyogenes* (21 – 23). For Gram-negative pathogens, proteins encoded within the heme transport operon have been studied, e.g., *Vibrio cholerae* (20). Based on the genome sequence, putative genes coding heme acquisition have been discerned in *V. cholerae* and called as Hut (heme utilization). Takeshi Uchida et al. (2012) have purified and characterized HutZ as a heme-degrading enzyme in *V. cholerae*, which is critical for heme utilization and can cleave heme to biliverdin *via* verdoheme (18). Therefore, heme can be degraded by HutZ and release iron to be used by bacteria. Besides, HutZ is also crucial for biofilm formation and contributes to the pathogenicity of *Edwardsiella piscicida* (24). In *Av. paragallinarum*, our previous research has also identified the preferential expression of HutZ in bacteria under iron-restriction condition based on RNA-seq analysis and qPCR, speculating that heme transport system can play an essential role in iron acquisition and HutZ protein plays an essential role in iron homeostasis of *Av. paragallinarum* (25). Nevertheless, the mechanism of interaction between HutZ and *Av. paragallinarum* remains unknown.

To gain further insight into the unknown mechanism of the function of HutZ in *Av. paragallinarum*, the HutZ mutant strain of *Av. paragallinarum* was constructed. Then, we further investigated the role of this protein on the growth, iron acquisition, heme utilization, and resistance to acidic stress in *Av. paragallinarum in vitro* and the effects of HutZ on the pathogenicity of *Av. paragallinarum in vivo*. So far, the interaction of *Av. paragallinarum* and macrophages has not yet been identified; therefore, the macrophages were used in *in vitro* study. To the best of our knowledge, this is the first report regarding the mechanism of the role of HutZ in *Av. paragallinarum* and will be of great significance for elucidating the mechanism of iron homeostasis and pathogenesis of *Av. paragallinarum* and exploring novel vaccines against *Av. paragallinarum*.

## RESULTS

### Expression and purification of HutZ

**Fig 1:**
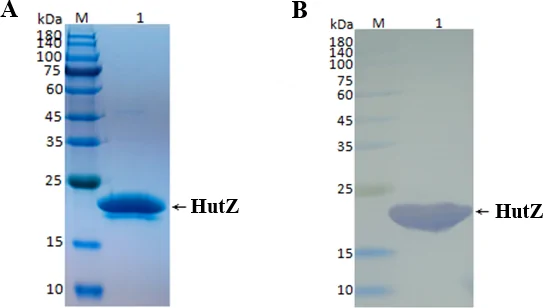
Identification of the HutZ antigen protein. (**A**) SDS-PAGE analysis. (**B**) Western blot analysis. M, marker. 1, purified His-tagged protein.

The HutZ gene was amplified, cloned into the pET22b vector, and expressed in *E. coli* receptor cells. Then, the expressed HutZ was purified. After cleavage of the His6-tag and size exclusion chromatography, HutZ protein was estimated to be >90% pure at a concentration of 2.24 mg/mL, and only one band was observed by SDS-PAGE with an apparent molecular mass of approximately 22 kDa (Fig. 1A), in agreement with calculated molecular mass for HutZ. Additionally, the purified HutZ antigen protein was confirmed by western blot analysis (Fig. 1B).

### Identification of HutZ mutant strain

**Fig 2:**
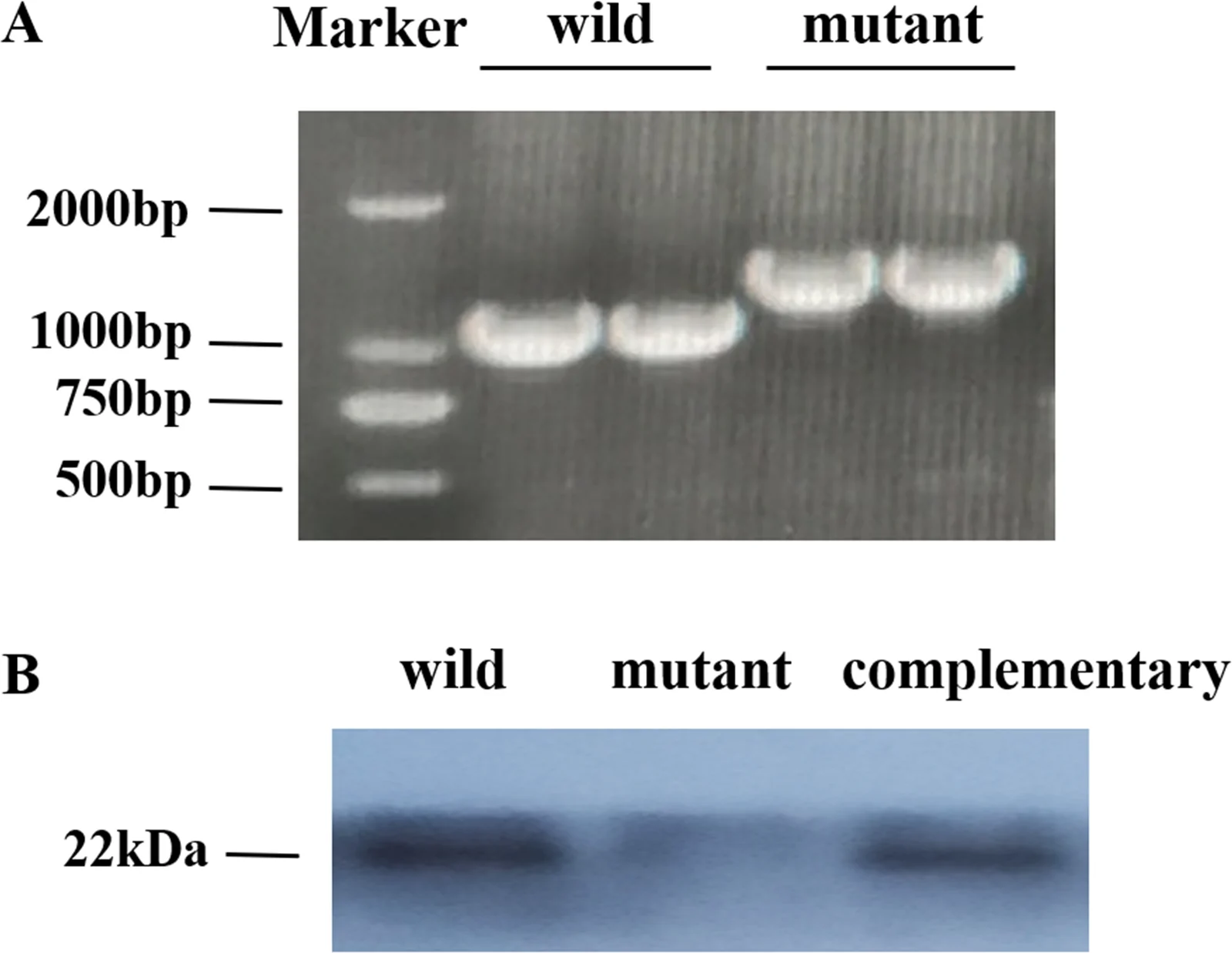
Identification of the HutZ mutant strain of *Av. paragallinarum*. (**A**) PCR analysis. (**B**) Western blot analysis.

Here, a mutant in which the HutZ gene was replaced by a Cm cassette was constructed. In order to evaluate whether the constructed HutZ mutant strain was successful, the strains were identified by PCR and western blot analysis. As shown in Fig. 2A, the HutZ gene was 519 bp in size, and the Cm cassette was 801 bp in size; thus, we could see that the product of the HutZ mutant strain was 282 bp larger than that of the wild-type strain. Thus, all of these sizes of fragments were also consistent with the expected fragments. Based on the results of western blot, there was higher protein expression of HutZ in the wild-type strain and HutZ complementary strain than in the HutZ mutant strain, which was 22 kDa in size as expected (Fig. 2B). No obvious protein band could be seen in HutZ mutant strain. Above all, the results demonstrate that the HutZ mutant strain is successfully constructed and can be applied in the following experiments.

### The effects of HutZ on the growth and iron acquisition of *Av. paragallinarum*

**Fig 3:**
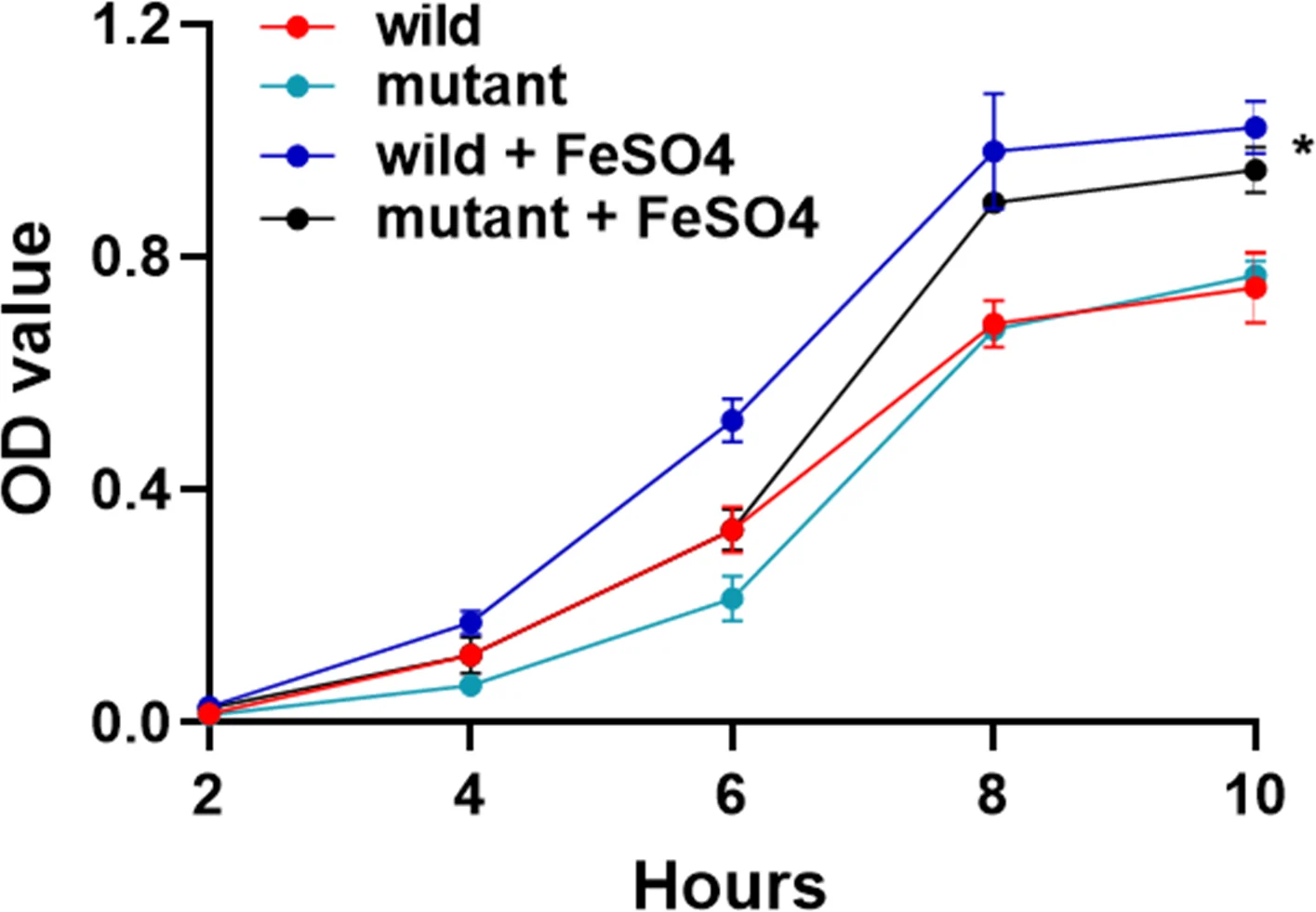
The effects of HutZ on the growth and iron acquisition of *Av. paragallinarum*. Cultures of the wild-type strain and HutZ mutant strain of *Av. paragallinarum* were diluted into MEMα medium containing FeSO4, and the growth was monitored. Graphs were shown from three independent replicates. *P < 0.05.

Since the HutZ mutant strain was constructed, we have detected the effects of HutZ on the growth of *Av. paragallinarum*. Here, we diluted the cultures of the wild-type strain and HutZ mutant strain of *Av. paragallinarum* into minimal essential medium alpha (MEMα) containing FeSO4, and the growth was monitored. As shown in Fig. 3, strains grew slowly in MEMα medium supplemented without iron, and two strains exhibited a comparable growth rate. However, all the strains grew better in MEMα medium supplemented with 40 µM iron. Under the iron condition, HutZ mutant strain grew more slowly than the wild-type strain at the same time, which showed a *P* < 0.05 at 10 h. Together, the results validate that HutZ can positively regulate the growth and iron acquisition of *Av. paragallinarum*.

### HutZ is needed for heme utilization in *Av. paragallinarum*

**Fig 4:**
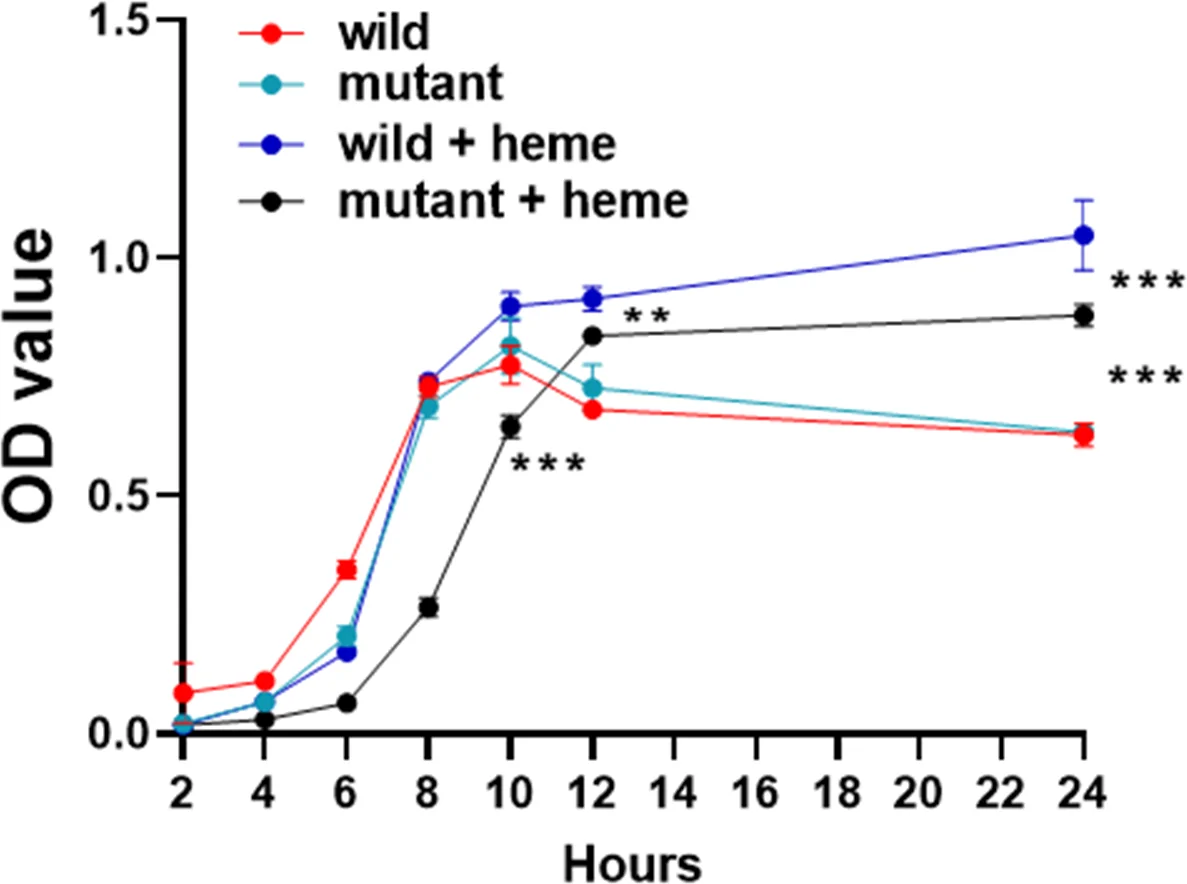
The effects of HutZ on heme utilization in *Av. paragallinarum*. Cultures of the wild-type strain and HutZ mutant strain of *Av. paragallinarum* were diluted into MEMα medium containing heme, and the growth was monitored. Graphs were shown from three independent replicates. **P < 0.01. ***P < 0.001.

To further investigate whether HutZ is required for heme iron utilization in *Av. paragallinarum*, we diluted the cultures of the wild-type strain and HutZ mutant strain of *Av. paragallinarum* into MEMα medium containing heme, and the growth was monitored. As seen in Fig. 4, heme could promote growth for both strains until 24 h. However, differences could be seen between the HutZ mutant strain and the wild-type strain in the case of additional heme. In the presence of heme, the HutZ mutant strain grew more slowly than the wild-type strain at the same time, which showed a *P* < 0.001 at 10 h. After a longer culture time, significant differences could also be seen at 12 h (*P* < 0.05) and 24 h (*P* < 0.001) between the two strains. Above all, the results demonstrate that HutZ is important for heme utilization in *Av. paragallinarum*.

### The effects of HutZ on bacterial resistance against acid stress

**Fig 5:**
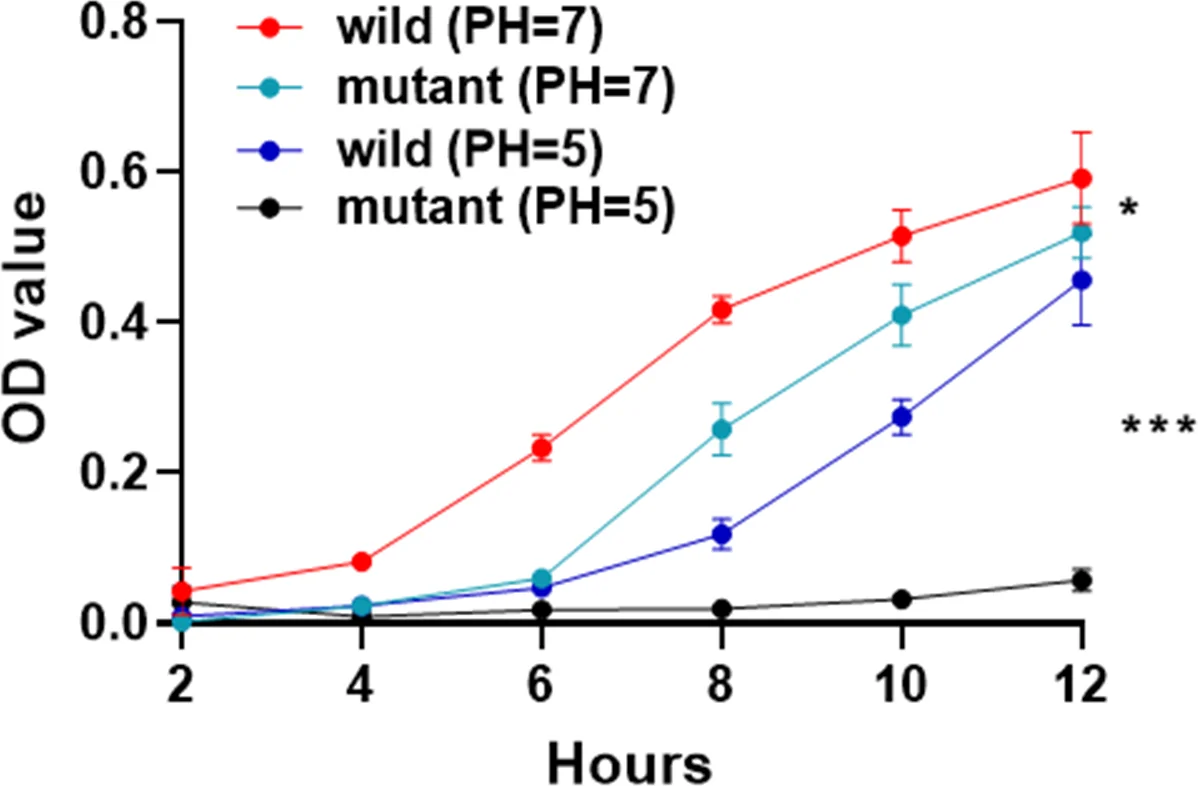
The effects of HutZ on bacterial resistance against acid stress. Cultures of the wild-type strain and HutZ mutant strain of *Av. paragallinarum* were diluted into TSB with 10% chicken serum and 0.0025% (wt/vol) reduced NAD under pH = 7 or pH = 5, and the growth was monitored. Graphs were shown from three independent replicates. *P < 0.05. ***P < 0.001.

Here, in order to verify whether HutZ participated in the stress resistance of *Av. paragallinarum*, we also detected the acid tolerance of the HutZ mutant strain. As displayed in Fig. 5, strains grew well when cultured under normal conditions, and the OD value of the wild-type strain was relatively higher than that of the HutZ mutant strain (*P* < 0.05). When cultured under acidic conditions, the HutZ mutant strain grew more poorly than the wild-type strain, and the OD value of the HutZ mutant strain was significantly lower than that of the wild-type strain at 12 h (*P* < 0.001). Thus, the results prove that HutZ in *Av. paragallinarum* can positively affect bacterial resistance against acid stress.

### HutZ involves in the pathogenicity of *Av. paragallinarum*

**Fig 6:**
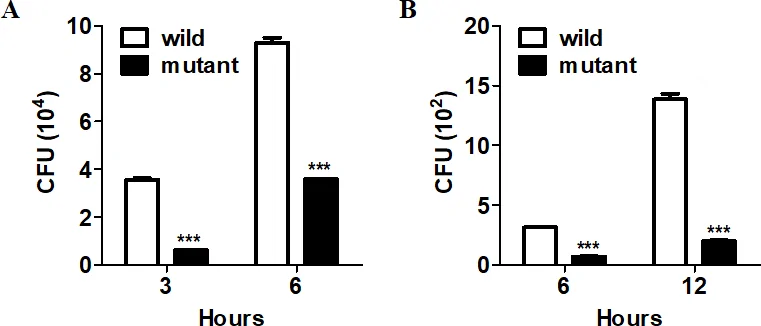
Pathogenicity tests of HutZ mutant strain *in vitro*. (**A**) *Av. paragallinarum* invasion of chicken macrophages HD11 cells that were infected with the same dose of mutant strain or wild-type strain for 3 h and 6 h. Then, cells were lysed, and the bacteria associated with and invaded HD11 cells were enumerated. (**B**) Replication of *Av. paragallinarum* in HD11 cells. After infecting with the mutant strain or wild-type strain for 6 h, cells were treated with cephalexin for 2 h. The cells were then incubated further for 6 h and 12 h, and the number of intracellular bacteria was determined by plate counting. Graphs were shown from three independent replicates. ***P < 0.001.

To evaluate the impact of HutZ on the pathogenicity of *Av. paragallinarum*, we assessed the role of HutZ mutant in *Av. paragallinarum* pathogenesis in both *in vitro* and *in vivo* infection experiments. In *in vitro* experiments to examine whether HutZ played any role in interaction with macrophages, cultured HD11 cells were incubated with HutZ mutant strain or wild-type strain, and the bacteria associated with the cells were enumerated. As seen in Fig. 6A, the amount of HutZ mutant bacteria recovered from the entire (i.e., from the surface and the intracellular milieu) HD11 cell culture was significantly lower than that of the wild-type after infecting for 3 h and 6 h, respectively (*P* < 0.001). To examine whether HutZ mutation played any role in the intracellular survival of bacteria, HD11 cells were incubated with HutZ mutant strain or wild-type strain for 6 h, and extracellular bacteria were killed. The cells were then incubated further for 6 h and 12 h, and the number of intracellular bacteria was also determined. The results showed that *Av. paragallinarum* is able to survive and replicate in macrophages. Besides, the number of intracellular HutZ mutant bacteria recovered from the cells was significantly lower than that of the wild-type at the same time points (*P* < 0.001) (Fig. 6B). Hence, the HutZ mutation significantly impaired the ability of *Av. paragallinarum* to adhere to and invade host cells.

**Fig 7:**
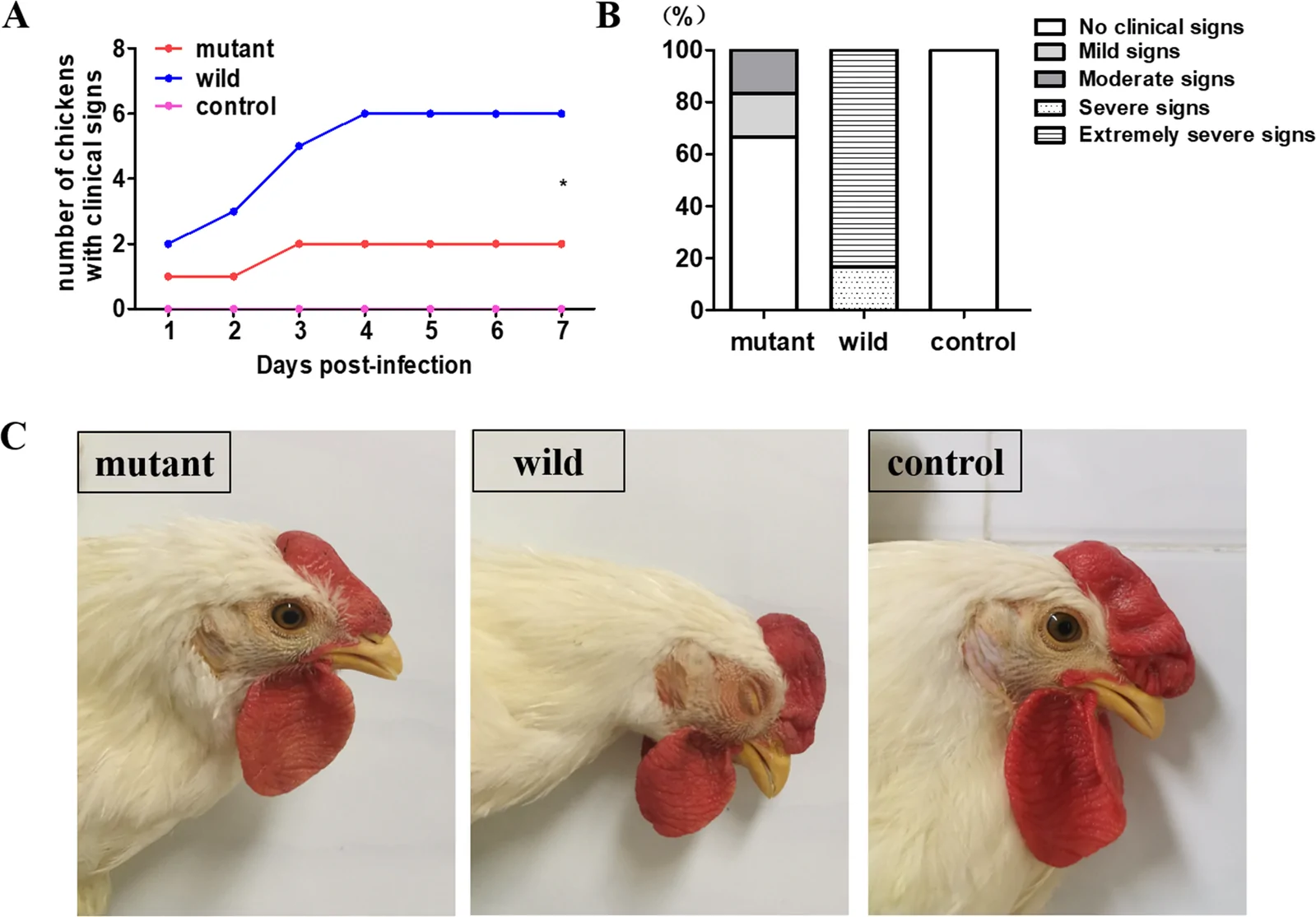
Pathogenicity tests of HutZ mutant strain *in vivo*. Chickens were challenged with 0.2 mL 5 × 10^4^ CFU/mL mutant strain or wild-type strain (*N* = 6). (**A**) The number of chickens with clinical signs was recorded. *P < 0.05. (**B**) The proportion of mild, moderate, severe, or extremely severe clinical signs of infected chickens was measured. (**C**) The clinical symptoms at day 5 post-infection were displayed.

Furthermore, the virulence of the HutZ mutant strain *in vivo* was investigated. First, we calculated and compared the ID50 of these strains after infection of diluted bacteria at different concentrations. By calculating the ID50, we found that the mutant strain had a dramatically increased ID50 (3.16 × 10^4^) compared to the wild-type strain (1.4 × 10^3^). Then, chickens were challenged with 0.2 mL 5 × 10^4^ CFU/mL mutant strain or wild-type strain, then the number of chickens with clinical signs and clinical symptoms was measured as shown in Fig. 7. Figure 7A showed that chickens infected with mutant strain had a significantly reduced number of diseased chickens (2/6) compared with chickens challenged with the wild-type strain (6/6) (*P* < 0.05). According to average scores for the clinical signs, the mutant strain induced milder clinical manifestations of IC in the chickens than the wild-type strain (Fig. 7B). The clinical signs of two chickens were scored as 1 and 2 in the mutant strain group, whereas the signs of five out of six chickens were scored as 3 and 4 in the wild-type strain group at 2–4 days after challenge. Figure 7C exhibits the clinical signs of chickens at 4 days following infection of two strains. In the mutant strain group, only slight changes were detected, such as facial swelling and nasal discharge. Nevertheless, the wild-type strain group showed much more severe damage, including facial swelling, nasal discharge blindness, and conjunctivitis. In the control group, no clinical signs of IC were seen, and the face remained its structure intact. Above all, HutZ is considered a key virulence factor and involved in the pathogenicity of *Av. paragallinarum*.

## DISCUSSION

Heme utilization systems play important roles in bacterial iron acquisition, adversity adaptation, and pathogenicity (24). Heme, an excellent iron source in the environment and hosts, can be used by bacteria in the free form or with heme-binding proteins. It is first transported into the cell with the assistance of the corresponding TonB-dependent outer membrane receptors and ATP-binding cassette transporter system proteins, and then, the iron is released from heme by cytoplasmic heme oxygenase. However, no heme oxygenase has been reported in some bacteria, e.g., *V. cholerae*. In 2004, HutZ was identified in *V. cholerae* by Wyckoff et al., which was regarded as a protein essential for the optimal utilization of heme as an iron source (26). In terms of *Av. paragallinarum*, BLAST searches against the NCBI database have also returned no heme oxygenase homologs for *Av. paragallinarum*. Therefore, the fate of heme after it enters *Av. paragallinarum*’s cytoplasm remains unknown, and little is known regarding which proteins contribute to heme utilization in the cytoplasm. Fortunately, we have also identified the HutZ protein in *Av. paragallinarum* based on RNA-seq analysis (25). Herein, we focused on the role of HutZ on growth and iron homeostasis in *Av. paragallinarum* in this study. This study will be helpful for realizing in-depth the molecular mechanism of the role of HutZ in *Av. paragallinarum* and might be of great significance for elucidating the mechanism of iron homeostasis and pathogenesis of *Av. paragallinarum* and exploring effective prophylactic vaccines against this kind of bacteria.

Here, we developed the HutZ mutant strain by replacing the HutZ gene with a Cm cassette to construct the recombinant plasmid. To evaluate whether our constructed mutant strain was successful, we identified them by PCR and western blot analysis. Not surprisingly, the PCR product of the HutZ mutant strain could get the fragments that were in line with expectations. To take western blot analysis, we expressed and purified HutZ antigen protein to be used for the preparation of anti-HutZ antiserum as the first antibody. Through the prokaryote expression system, the coding sequences of the HutZ gene were expressed in and purified from *E. coli*, which exhibited a molecular mass comparable to that predicted for HutZ (~22 kDa). In the western blot analysis of the mutant strain, we also compared the protein expressions of HutZ in the mutant strain with the wild-type strain as well as the constructed complementary strain. The complementary strain was developed by cloning the amplified HutZ gene into the pBBR1MCS-2 vector to construct the recombinant plasmid. As expected, the HutZ protein could be highly expressed in the wild-type strain and HutZ complementary strain, whereas the mutant strain showed no expression of this protein. Hence, the HutZ mutant strain of *Av. paragallinarum* was successfully constructed.

As iron is indispensable for the growth of bacteria, we assessed the role of HutZ on the growth and iron acquisition of *Av. paragallinarum*. When incubated in MEMα medium containing FeSO4, the wild-type strain grew well compared with that in iron-restriction condition, which was in line with the results of our previous study (25). Notably, the HutZ mutant strain had a lower growth rate than the wild-type strain under iron conditions, indicating HutZ can have a regulatory role in the growth and iron acquisition of *Av. paragallinarum*. Due to this important function of HutZ, it is meaningful to deeply explore the regulating mechanism behind these results.

Most heme-transporting organisms can use heme as an iron source, since heme promotes their growth on iron-restricted media. In a few pathogenic bacterial species, including *Staphylococcus aureus*, *Neisseria meningitidis,* and *Pseudomonas aeruginosa*, heme oxygenase has been identified to be an enzyme that removes the iron from heme moiety (27 – 29). However, no heme oxygenase activity has been identified in some other bacteria, such as *Vibrio cholerae*. Wyckoff and coworkers have found that a novel heme utilization protein, HutZ, is required for optimal heme utilization in *Vibrio cholerae*, which has the properties expected of a heme carrier or storage protein (26). However, it seems that the function of HutZ can differ depending on the species of bacteria. For example, a previous study has demonstrated that HutZ is not required for heme utilization in *Edwardsiella piscicida*, and the growth of both TX01ΔhutZ and TX01 exhibited a similar trend with no significant difference with the increase in heme concentration (24). Here, in order to examine whether HutZ is a key factor involved in heme utilization in *Av. paragallinarum*, we compared the growth of the HutZ mutant strain and wild-type strain in an iron-restricted medium with heme as the sole iron source. Just like *Vibrio cholerae*, the growth of the HutZ mutant strain was extremely poorer in this situation compared with the wild-type strain. In consequence, HutZ is an important factor for heme utilization in *Av. paragallinarum*, which is needed for growth at low iron concentrations following growth with heme as the sole iron source. Here, it seemed that the HutZ mutant strain grew better with heme than without it, and we speculate that some other protein components might also involve in heme utilization in *Av. paragallinarum*. As the HutZ protein role becomes essential in heme utilization for *Av. paragallinarum*, it is worthy to further explore the mechanism of HutZ in the maintenance of iron homeostasis.

Bacteria are adapted for efficient functioning under normal physiological environments. Extreme variation from the optimum environmental conditions stresses the bacteria resulting in inhibition of its growth, an increase in its lag time with reduced growth rate, or its ultimate death (30, 31). Acid tolerance is an important trait for various pathogens during infection and is regulated by the regulator Fur in a variety of pathogens, such as *E. coli*, *Salmonella typhimurium*, and *Aeromonas salmonicida* (32 – 34). The ability of bacteria to tolerate acid stress plays an important role in their growth and survival (35). Acid resistance is an indispensable mechanism for the survival of neutralophilic bacteria, and a change in pH scale in growth conditions is the primary stress for most neutralophilic bacteria, such as *E. coli* (36). Here, we also investigated whether HutZ possesses the function of adversity resistance. We found that the HutZ mutant strain grew poorly at a pH as low as 5, which was consistent with the results of previous research by *Shi* et al. (24). Therefore, mutation of HutZ markedly attenuates the acid tolerance capability of *Av. paragallinarum*, suggesting that HutZ has a positive regulatory role on *Av. paragallinarum* to be adaptable to the adversity environment of acid stress.

Several factors are responsible for the pathogenicity of *Av. paragallinarum*. Among them, hememagglutinin has been regarded as the key virulence factor because it participates in tissue adhesion (37). Heme utilization systems also play an important role in bacterial pathogenicity. For example, previous research has confirmed that HutZ contributes to the pathogenicity of *Edwardsiella piscicida*, which inactivation of HutZ significantly impairs the ability of bacteria to invade and reproduce in host cells and infect host tissue (24). In our study, we also aimed to investigate whether HutZ contributed to the pathogenicity of *Av. paragallinarum in vitro* and *in vivo*. To date, the interaction of *Av. paragallinarum* and macrophages has not been reported. Here, we demonstrated for the first time that *Av. paragallinarum* can survive and replicate in chicken macrophages. In addition, HutZ mutation significantly weakened the ability of *Av. paragallinarum* to invade host macrophages and declined the capability of *Av. paragallinarum* to survive and replicate in macrophages. By *in vivo* challenge of the HutZ mutant strain in chickens, we validated that this mutant strain had significantly lower pathogenicity for chickens under the same challenge dosages compared with the wild-type strain, displaying low morbidity and mild clinical symptoms. Besides, the ID50 value of the mutant strain was also higher than the wild-type strain. Therefore, HutZ can be considered a novel virulence factor related to the pathogenicity of *Av. paragallinarum*, which can be helpful to search for new strategies to fight against infectious coryza by targeting this protein.

In conclusion, we present here the first characterization of HutZ from *Av. paragallinarum*. Our results show that HutZ can not only involve in iron acquisition and heme utilization but also plays an important role in coping with adverse circumstances including acid stress. It can function as a virulence factor that is essential to bacterial infection in *in vitro* chicken macrophages and the live chicken model. These observations demonstrate for the first time the essential roles of HutZ on iron homeostasis and pathogenesis of *Av. paragallinarum*, and this gene is the potent novel candidate target that should be tested in various vaccine platforms and can be beneﬁcial in the prophylaxis of *Av. paragallinarum* infection.

## MATERIALS AND METHODS

### Bacterial strain and growth conditions

The strain 3005 of *Av. paragallinarum* serogroup C was used in this study, which was isolated from China in 2018. Tryptic Soy Broth (TSB) and Tryptic Soy Agar (TSA) that were added with 10% chicken serum and 0.0025% reduced nicotinamide adenine dinucleotide (NAD) were used for propagation and maintenance of the strain.

### Expression and purification of HutZ antigen protein

The HutZ antigen protein was expressed and purified *via* prokaryotic expression system by Sangon Biotech (Shanghai, China). Briefly, the plasmid was constructed by whole gene synthesis and subcloned into pET22b expression vector. The recombinant HutZ protein was further transformed into *E. coli* receptor cells BL21(DE3), cultured, induced, collected, and purified to the expected concentration of 2.24 mg/mL. Protein purity was assessed by sodium dodecyl sulfate−polyacrylamide gel electrophoresis (SDS-PAGE) on 12% polyacrylamide gels as previously reported and by western blot as described below (38).

### Construction of HutZ mutant and complementary strain

For the construction of the HutZ mutant strain, the HutZ gene and its upstream fragment at about 1,000 bp and its downstream fragment at about 1,000 bp were amplified from the genomic DNA of *Av. paragallinarum* and cloned into the pGEM-T Easy vector between the NdeI and NcoI restriction cut sites, in which HutZ gene was replaced by a chloroamphenicol (Cm) cassette to construct the recombinant plasmid. *E. coli* strains transduced with recombinant plasmid were grown at 37°C in LB broth supplemented with 50 ng/µL ampicillin. Then, 1 µL of recombinant plasmid at a concentration of 700 ng/µL was electrotransformed into 65 µL competent cell of strain 3005 and incubated at 900 µL SOC for 1 h at 37°C. Finally, the bacteria in SOC were centrifuged and cultured in TSA plate supplemented with 2 µg/mL Cm. The bacterial colony grown on the plate was determined by PCR, and the procedures could be referred to as previously described (39). The primer sequences were as follows: F: 5′- AAAGTGAGCGTGGCTTATCAATTCT-3′; R: 5′- AAGCTCACACATAAGGCTGAGTAATAAAT-3′. Positive clones were incubated in TSB supplemented with 2 µg/mL Cm and stored at −80°C for further experiments.

For the construction of the HutZ complementary strain, the HutZ gene was amplified from the genomic DNA of *Av. paragallinarum* and cloned into the pBBR1MCS-2 vector between the XbaI and XhoI restriction cut sites to construct the recombinant plasmid. *E. coli* strains transduced with recombinant plasmid were grown at 37°C in LB broth supplemented with 50 ng/µL kanamycin (Km). Then, 2 µL recombinant plasmid at a concentration of 140 ng/µL was electrotransformed into 65 µL competent cell of HutZ mutant strain and incubated at 900 µL SOC for 1 h at 37°C. Finally, the bacteria in SOC were centrifuged and cultured in TSA plate supplemented with 20 ng/µL Km. Bacterial growth from the plate was incubated in TSB supplemented with 20 ng/µL Km and stored at −80°C for further experiments.

### Preparation of anti-HutZ antiserum

The HutZ antigen protein was mixed with an immune adjuvant of QuickAntibody-Mouse3W (KX0210042) that was obtained from Biodragon Technology Co., Ltd (Suzhou, China) at 1:1 and was then intramuscularly immunized into the hind leg of BALB/c mouse (6 wk of age) at a dose of 50 µg/mouse two times, with an interval of 14 days. The negative control mice were also used, which received only the same volume of sterile saline. On day 7, after the second immunization, tail blood was collected to measure the antibody titer. Mice that had high antibody titers were sacrificed and blood was gained.

### Western blot analysis

HutZ mutant and complementary strain were further identified by western blot analysis. Bacterial cells were centrifuged at 10,000 × *g* for 10 min, quantified using the BCA protein assay (Beyotime, Beijing, China), resolved on 12% polyacrylamide gels, and transferred to polyvinylidene fluoride nylon membrane (Millipore, USA). The membrane was then incubated with mouse anti-HutZ polyclonal antibody as the first antibody and goat anti-mouse IgG polyclonal antibody as the second antibody. Antibody binding was detected using a Western Lightning Chemiluminescence kit (Thermo, USA). Notably, if western blot analysis was adapted for the identification of purified HutZ antigen protein, mouse anti-His tag was used as the first antibody, and goat anti-mouse IgG polyclonal antibody was used as the second antibody.

### Detection of growth under the iron condition

The wild-type strain 3005 of *Av. paragallinarum* and HutZ mutant strain were grown under *in vitro* culture conditions with iron restriction or iron repletion as previously described (25). First, two strains were incubated in TSB and grown in a shaking incubator at 37°C until reaching an optical density (OD) of 0.6 at 600 nM (exponential phase of growth). For the iron-restriction conditions, bacterial strains were inoculated at 1:100 in 6 mL MEMα (Invitrogen, USA) with 0.0025% (wt/vol) reduced NAD. For the iron-repletion conditions, an extra 40 µM FeSO4 was added in cultures based on the related reference. The cells were grown in a shaking incubator at 37°C, and OD values were measured every 2 h. Three replicates were made under each condition.

### Heme utilization assay

Heme utilization assay was performed just like that of detection of growth under iron conditions. Briefly, cultured wild-type strain and HutZ mutant strain at the same OD were inoculated at 1:100 in 6 mL MEMα (Invitrogen, USA) with 0.0025% (wt/vol) reduced NAD, and extra 5 µM hemin was added to the cultures. Then, the cells were grown in a shaking incubator at 37°C, and OD values were measured every 2 h. Three replicates were made under each condition.

### Resistance to acid stress

To determine acid tolerance, cultured wild-type strain and HutZ mutant strain at the same OD were inoculated at 1:100 in 6 mL TSB with 10% chicken serum and 0.0025% (wt/vol) reduced NAD under pH = 7 or pH = 5. The cells were grown in a shaking incubator at 37°C, and OD values were measured every 2 h. Three replicates were made under each condition.

### Pathogenicity tests of HutZ mutant strain *in vitro*

To assess the virulence and pathogenicity of the HutZ mutant strain in macrophages, the chicken macrophage HD11 cells were used in this study and were cultured as described previously (40). Then, HD11 cells were preincubated in 12-well plates and mixed with HutZ mutant strain and wild-type strain at a multiplicity of infection of 10:1. After incubation at 37°C for 3 h and 6 h, the plates were washed three times with phosphate-buffered saline (PBS). To determine the number of bacterial cells associated with the entire HD11 cells, the washed HD11 cells were lysed with 1 mL of 1% (vol/vol) Triton X-100 in PBS, and the number of bacteria was counted by dilution plating. To determine the number of bacterial cells that had penetrated HD11 cells, the above-mentioned washed HD11 cells were incubated with cephalexin (50 µg/mL) for 2 h to kill extracellular bacteria. After washing three times with PBS, the cells were incubated for 6–12 h. HD11 cells were lysed and plated as described above.

### Pathogenicity tests of HutZ mutant strain *in vivo*

To assess the virulence and pathogenicity of the HutZ mutant strain in chickens, the HutZ mutant strain and wild-type strain were diluted to 5 × 10^5^ CFU/mL, 5 × 10^4^ CFU/mL, and 5 × 10^3^ CFU/mL, respectively. A total of 42 specific-pathogen-free (SPF) White Leghorn chickens (9 wk of age) were randomly allocated into seven groups of six chickens each. Then, chickens were challenged by infraorbital sinus inoculation with 0.2 mL of the HutZ mutant strain or the wild-type strain at different concentrations separately. Chickens in the control group were treated with PBS alone. Clinical signs of infectious coryza were recorded from day 1 to day 7 post-infection. The presence and degree of nasal discharge and facial swelling in the challenged chickens were scored according to the following scale, similar to the previously reported studies; specifically, 0: no clinical signs; 1: mild signs (slight facial swelling); 2: moderate signs (moderate facial swelling and nasal discharge); 3: severe signs (severe facial swelling, abundant nasal discharge, lacrimation, and partially closed eye); 4: extremely severe signs (extremely severe facial swelling, abundant nasal discharge, lacrimation, and complete closure of the eye) (41). Finally, the 50% infective dose (ID50) of each strain was calculated by the improved Kärber method formula (42).

Subsequently, a total of 18 SPF White Leghorn chickens (9 wk of age) were randomly allocated into three groups of six chickens each. Then, chickens were challenged by infraorbital sinus inoculation with 0.2 mL of the HutZ mutant strain or the wild-type strain at 5 × 10^4^ CFU/mL separately. Chickens in the control group were treated with PBS alone. Then, morbidity and clinical symptoms were measured as mentioned above.

### Animals and ethics statement

We carried out the animal experiments in strict accordance with the requirements of the Laboratory Animal Requirements of Environment and Housing Facilities (GB14925-2010, National Laboratory Animal Standardization Technical Committee) and the Chinese Regulations of Laboratory Animals Guidelines (Ministry of Science and Technology of People’s Republic of China). All experimental procedures were approved and audited by the Beijing Academy of Agriculture and Forestry Sciences Animal Care and Use Committee guidelines [ID: SYXK (Jing) 2017–0039], which were approved by the animal welfare committee of Beijing Academy of Agriculture and Forestry Sciences (15 December 2017).

### Data analysis

Data analysis was conducted using a two-way analysis of variance with GraphPad Prism (ver. 5.0). *P* < 0.05 represents statistical significance. The results were shown as the means ± standard deviations of three independent experiments.

## Data Availability

All relevant data were within the manuscript.
